# Pplase of *Dermatophagoides farinae* promotes ovalbumin-induced airway allergy by modulating the functions of dendritic cells in a mouse model

**DOI:** 10.1038/srep43322

**Published:** 2017-02-27

**Authors:** Hui Wang, Lihua Mo, Xiaojun Xiao, Shu An, Xiaoyu Liu, Jinge Ba, Weifang Wu, Pixin Ran, Pingchang Yang, Zhigang Liu

**Affiliations:** 1 State Key Laboratory of Respiratory Disease for Allergy at Shenzhen University, Shenzhen Key Laboratory of Allergy & Immunology, Shenzhen University School of Medicine, Shenzhen, China; 2 Shenzhen ENT Institute, Longgang ENT Hospital, Shenzhen, China; 3 Luohu district people’s hospital, Shenzhen, China; 4 State Key Laboratory of Respiratory Disease, Guangzhou Medical University, Guangzhou 510006, China

## Abstract

Our previous studies revealed that many proteins in addition to the known allergens of *D. farinae* have not been fully characterized. We observed that Pplase did not respond to serum collected from patients sensitized to *D. farinae*. In a mouse model, Pplase significantly enhanced airway hyperresponsiveness (AHR) and Th2 responses induced by ovalbumin (OVA) compared with mice treated with OVA alone. Moreover, exposure to Pplase significantly increased the expression of IRF4, CD80, CD83, MHCII and TNF-α in DC2.4 cells, which was abolished in the presence of a TLR4 inhibitor. *In vitro* T cell polarization experiments revealed that Pplase alone could not induce T cell polarization but enhanced T cell polarization together with OVA. In addition, transfer of Pplase-primed bone marrow-derived DCs (BMDCs) to naïve mice enhanced AHR and Th2 immune responses in mice sensitized to OVA. In conclusion, Pplase is not an allergen of *D. farinae* but can activate DC cells to facilitate OVA-induced allergic responses.

Asthma is a common disease. The incidence of allergic diseases worldwide is approximately 1–18%^1^. Dust mites produce the most significant inhalant allergens. It has been reported that nearly 80% of asthma patients are sensitized to dust mite allergens^2^,^3^. *Dermatophagoides pteronyssinus* (Der p) and *Dermatophagoides farinae* (Der f) are the most important indoor species of dust mites^4^. Previously published data indicate that *D. farinae* contains Der f 1–23 subtypes of allergens, and Der f 1 and 2 are the major allergens^5^. Although there are many allergens in the air, such as those found in dust mites, pollen, cockroaches, fungi and animal feathers, more than 70–80% of asthma patients are sensitized to dust mites, while less than 40% asthma patients are sensitized to other airborne allergens, suggesting that there may be some unknown mechanisms by which dust mites facilitate the development of allergic diseases^6^.

Imbalance of Th1 and Th2 response is considered the major pathogenesis of allergic disease^7^,^8^. Dendritic cells (DCs) capture allergens and present allergen information to T cells^9^. There are a number of pattern recognition receptors (PRRs), such as Toll-like receptors (TLRs), on the surface of DCs that recognize microbial products^10^. Allergen-primed DCs activate naive T cells to differentiate into subsets of effector T cells (i.e., Th1 or Th2) via MHC II-allergen peptide complexes, cytokines, and costimulatory molecules^11^. It is accepted that DCs are the most important antigen-presenting cells (APCs) and play a critical role in the pathogenesis of allergic asthma^9^,^12^. Conditional deletion of interferon regulatory factor 4 (IRF4) in CD11c cells revealed a reduction of Th2 responses induced by house dust mites (HDMs) in mouse models, while upregulation of IRF4 expression in BMDCs can drive more T cells toward differentiation into a Th2 subset^13^,^14^. Thus, when studying the mechanisms underlying allergen-induced diseases, it is important to understand the role of DCs.

In previous studies, we analyzed the genome and transcriptome of dust mites using high-throughput sequencing and bioinformatics and identified many other proteins in addition to Der f 1–23 in *D. farinae*^15^. By performing homologous alignment, we identified a protein showing very high homology to the phthalein proline cis/trans isomerase peptidyl-prolylisomerases (Pplase) of *Tachypleus tridentatus and* designated this protein *D. farinae* Pplase. It has been reported that *Helicobacter pylori* Pplase stimulates the secretion of Th17^16^. Further studies showed that Pplase could activate p38 MAPK and caspase-8 in gastric epithelial cells by activating TLR4 and subsequently induces apoptosis of gastric epithelial cells^17^. Taken together, these findings suggest that the Pplase protein of *D. farinae* may be associated with the pathogenesis of allergic diseases. However, the functions of *D. farinae* Pplase are unclear. Here, we aim to determine the role of *D. farinae* Pplase in the development of airway allergy in a mouse model.

## Materials and Methods

### Chemicals

A peroxidase-labeled mouse anti-human IgE Fc antibody and peroxidase-labeled goat anti-mouse IgE, IgG1 and IgG2a Fc antibodies were obtained from SouthernBiotech, USA (9160-05, 1110-05, 1070-05 and 1155-05); tetramethylbenzidine (TMB) was purchased from Solarbio, China (R1200); aluminum hydroxide was obtained from Thermo Fisher, USA (77161); and LPS was purchased from Sigma, USA (L3012). ELISA kits for IL-4, IFN-γ and TNF-α were obtained from Ebioscience, USA (88-7044, 88–7314 and 88–7324); ELISA kits for IL-5 and IL-13 were purchased from 4 A Biotech, China (CME0003, CME0009); an IRF4 antibody was obtained from Cell Signaling, USA (4964); an antibody against GAPDH was procured from Proteintech, China (10494-1-AP); a TLR4 signaling inhibitor was purchased from Invivogen, USA (CLI-095); an anti-mouse TLR2 Ab was obtained from Biolegend, USA (121802); the PE-CD80, PE-CD83, FITC-MHCII and FITC-CD40 antibodies were obtained from Ebioscience, USA (12–0801, 12–0831, 11–5321 and 11–0402); and mouse GM-CSF and IL-4 were purchased from Sino Biological, China (51048-M07H, 51084-M08B). Anti-CD3 and anti-CD28 antibodies were obtained from Ebioscience, USA (16-0031-82, 16-0281-82).

### Preparation of recombinant Pplase protein

*Pplase* PCR products were ligated into the pMD19-T vector (Takara), followed by transformation into *E. coli* Top10 cells. Plasmids from positive clones were digested with BamHI and HindIII. The target fragment was ligated into PET-32a and then transformed into *BL21* for expression. Bacteria were grown in LB broth supplemented with 50 μg/ml ampicillin. After induction using isopropyl-D-thiogalactopyranoside (IPTG), the bacteria were incubated for 4 h at 37 °C and then harvested and resuspended in 50 mM Tris–HCl, 100 mM NaCl, pH7.5 for sonication. Pplase proteins were purified via affinity chromatography. The endotoxin was preliminarily removed using an ion exchange column and further eliminated using the ToxinEraser Endotoxin Removal Kit (L00338, Genscript, China). The LPS concentration tested using the ToxinSensor Chromogenic LAL Endotoxin Assay Kit (L00350C, Genscript, China) was lower than 0.1 EU/ml.

### Assessment of Pplase allergenicity

Skin prick test (SPT) of Pplase: The endotoxin was removed from the Pplase solution (0.01 mg/ml). Histamine phosphate (0.1%) and saline were used as a positive control and negative control, respectively. The allergenicity of Pplase was tested in 10 patients sensitized to HDMs. The scoring of the results was as follows: 4+: the response was stronger than the histamine control; 3+: the response was nearly the same as the histamine control; 2+: the response was weaker than for histamine, but stronger than the negative control; 1+: the response was significantly weaker than for histamine, but slightly stronger than the negative control; negative: no response. In the present study, written informed consent was obtained from each human subject. The experimental procedures were approved by the Human Ethics Committee at Shenzhen University, and all procedures were performed according to the required guidelines.

### Enzyme-linked immunosorbent assay (ELISA)

Specific serum IgE antibodies for Pplase were measured using ELISA. Briefly, the ELISA microtiter plates were coated with 100 ng of Pplase per well and incubated at 4 °C overnight, and then blocked with 200 μl of 5% bovine serum albumin (BSA) diluted in PBS at room temperature. After 1 h, serum was added to each well (100 μl/well), followed by incubation for 2 h and subsequent incubation with peroxidase-labeled goat anti-human IgE (1:2000) for 1 h at room temperature. After each step, washing with PBST was performed 3 times. Development was induced by adding tetramethylbenzidine (TMB; 100 μl/well) and terminated with 2 M H_2_SO_4_ (50 μl/well). The plates were finally read using an ELx808 absorbance microplate reader (BioTek, Shanghai, China) at 450 nm.

### Induction of allergic airway inflammation

Female BALB/c mice (6–8 weeks) were purchased from the Guangdong Experimental Animal Center, and the mice were maintained in a pathogen-free facility at Shenzhen University. Experimental procedures were approved by the Animal Ethics Committee at Shenzhen University. All procedures were performed according to the required guidelines. As shown in Fig. S1A, mice were immunized intraperitoneally with a mixture of Pplase (100 μg/mouse) and OVA (100 μg/mouse) or with Pplase (100 μg/mouse) or OVA (100 μg/mouse) alone, in 0.1 mL of 2% aluminum hydroxide, on days 0, 3, and 7, as described elsewhere. PBS was used as a negative control. The mice of OVA and Pplase/OVA groups were subsequently challenged with OVA (50 μg/mouse) via nostril drop administration for one week, and the mice of Pplase groups were subsequently challenged with Pplase (50 μg/mouse) in 50 μl of PBS via nostril drop administration for one week. On day 22, airway resistance was measured via the inhalation of incremental doses of methacholine (0, 6.25, 12.5, 25, 50 and 100 mg/ml). The mice were euthanized on day 23, and lung tissues were fixed in 4% formalin, then embedded in paraffin wax for further analysis. Bronchoalveolar lavage fluid (BALF) was collected in 1 ml of PBS, and the total cells were stained with Giemsa stain and quantified using a microscope.

### Cytokine ELISA, immunoglobulin assays and neutralization of TLR2 and TLR4 receptors

Splenocytes (5 × 10^6^ cells per well) were incubated in enriched medium for 48 h, and the levels of IL-4, IL-5, IL-13 and IFN-γ in the culture medium and BAL fluid were determined via sandwich ELISA with commercial reagent kits (Ebioscience) according to the manufacturer’s instructions. The levels of specific IgE, IgG1 and IgG2a in mouse serum were also assessed through indirect ELISA.

DC2.4 cells were cultured at 37 °C under 5% CO_2_ in Dulbecco’s modified Eagle’s medium (DMEM) supplemented with 10% fetal bovine serum and 10 mM HEPES. Prior to the neutralization experiment, DC2.4 cells (a murine macrophage cell line) were seeded into 96-well plates (0.5 × 10^6^ cells/well) for 20 hours. DC2.4 cells were treated with an anti-mouse TLR2 Ab or TLR4 signaling inhibitor for 120 min or 6 h at 37 °C, respectively, and then stimulated with OVA, Pplase and Pplase/OVA. At 24 h post-stimulation, cell-free supernatants were collected, and cytokine production was determined via ELISA according to the manufacturer’s instructions (Ebioscience).

### Quantitative real-time PCR (qRT-PCR)

Determination of the expression levels of IRF4, TNF-α, TLR2, TLR4, ERK, JNK, P38, NFκB1 and NFκB2 was achieved through qRT-PCR. Briefly, DC2.4 cells were seeded into a 6-well dish at a density of 5 × 10^5^ cells per well and maintained at 37 °C in 5% CO_2_. After overnight growth, the DC2.4 cells were stimulated with Pplase or OVA at a concentration of 20 μg/ml. At 4 hours after stimulation, the medium was removed, and 200 μl of trypsin was added at 37 °C. After 5 minutes, 800 μl of C-DMEM was added per well, and the mixture was then transferred to a clean EP tube. The samples were centrifuged at 1500 rpm. RNA extraction was performed according to the instructions of the RNA extraction kit (Fastagen, China, 220010), and the concentration was calculated at OD260. A total of 100 μg of RNA was used for reverse transcription and RT-PCR (Transgen, China, AT341). The following primers specific for each gene were employed in this study: IRF4, 5′-ACAGCACCTTATGGCTCTCTG and 3′-ATGGGGTGGCATCATGTAGT^18^; TLR4, 5′-TGCCTTCACTACAGGGACTTT and 3′-TGGGACACCACGACAATAAC^19^; TLR2, 5′-TGTGCCACCATTTCCACG and 3′-AAAGGGCGGGTCAGAGTT^20^; ERK, 5′-GCTCTGCCCTATTTCATCTTGT and 3′-ATCCAATCACCCACACACAG^19^; JNK, 5′-CTCCAGCACCCATACATCAAC and 3′-TCAGTTCTTTCCACTCCTCTATTG^19^; P38, 5′-ACACATCCAACAGACCAATCAC and 3′-ATTTCCACGATTTCCCAGAGA^19^; NFκB1, 5′-CAGCTCTTCTCAAAGCAGCA and 3′-TCCAGGTCATAGAGAGGCTCA^21^; NFκB2, 5′-TGGAACAGCCCAAACAGC and 3′-CACCTGGCAAACCTCCAT^21^; GAPDH, 5′-CATGGCCTTCCGTGTTCCTA and 3′-GCGGCACGTCAGATCCA^22^. Primers specific for GAPDH were used as an endogenous control for sample normalization, and the cDNA employed in these experiments was obtained from three independent replicates.

### Flow cytometry

Prior to stimulation, DC2.4 cells were seeded into a 6-well dish at a density of 2 × 10^5^ cells per well and maintained at 37 °C under 5% CO_2_. After overnight growth, DC2.4 cells were treated with or without a TLR4 signaling inhibitor for 6 h at 37 °C and then stimulated with Pplase or LPS or/and OVA at a concentration of 20 μg/ml. At 24 hours after stimulation, the medium was removed, and 200 μl of trypsin was added at 37 °C. After 5 minutes, 800 μl of C-DMEM was added per well, and the mixture was then transferred to a clean EP tube. The samples were then centrifuged at 1500 rpm, after which the medium was removed, and CD80, CD83, MHCII and CD40 antibodies (Ebioscience) were added, followed by incubation in the dark for 2 hours. Next, the samples were centrifuged at 1500 rpm, and the supernatant was removed; this procedure was repeated 3 times. Finally, 500 μl of PBS was added, followed by mixing and transfer to a flow tube for flow cytometric analysis.

### Bone marrow-derived DC (BMDC) generation, *in vitro* T cell priming and polarization

BMDCs were generated as previously described^23^. Briefly, bone marrow cells were obtained from the femurs and iliac bones of female BALB/c mice and placed in RPMI 1640 medium containing 10% FBS, 100 U/ml penicillin/streptomycin, 20 ng/ml recombinant mouse GM-CSF and IL-4. One-half of the volume was replaced every other day. On day 8, all cells were recovered by trypsin digestion.

Splenocytes were harvested by mincing spleen tissue and passing the cells through a nylon mesh sieve. The cells were then cultured in RPMI 1640 medium containing 10% FBS. Prior to the T cell differentiation assays, 96-well plates were coated with an anti-CD3 antibody (1 μg/ml), then maintained at 4 °C overnight and washed twice with PBS. Next, BMDCs (2 × 10^4^) were seeded into 96-well plates and pre-stimulated with Pplase (10 μg/ml or 20 μg/ml) or LPS (1 μg/ml) for 24 hours, then washed three times with PBS. Subsequently, splenocytes (6 × 10^5^) were co-cultured with BMDCs in the presence of OVA (20 μg/ml) and anti-CD28 (2 μg/ml) for 5 days, and the supernatants were collected to detect cytokine levels according to the manufacturer’s instructions.

### Transfer of Pplase-primed BMDCs

BMDCs were cultured in the presence or absence of TLR4 signaling inhibitors for 6 hours and then stimulated with Pplase (10 μg/ml or 20 μg/ml) or LPS (1 μg/ml) for 24 hours. The samples were subsequently washed three times with PBS, after which these BMDCs (1 × 10^6^ cells) were injected into BALB/c mice i.v. just before OVA priming. After the transfer of the BMDCs, the mice were sensitized and challenged with OVA, as described above (Fig. S1B).

### Data statistics

The data (mean ± SEM) were processed using GraphPad software, and significant differences between two groups were tested using the unpaired two-tailed t-test. *P < 0.05. **P < 0.01. ***P < 0.001. ns, no significant difference.

## Results

### Pplase of *D. farinae* is not an allergen

**Figure 1:**
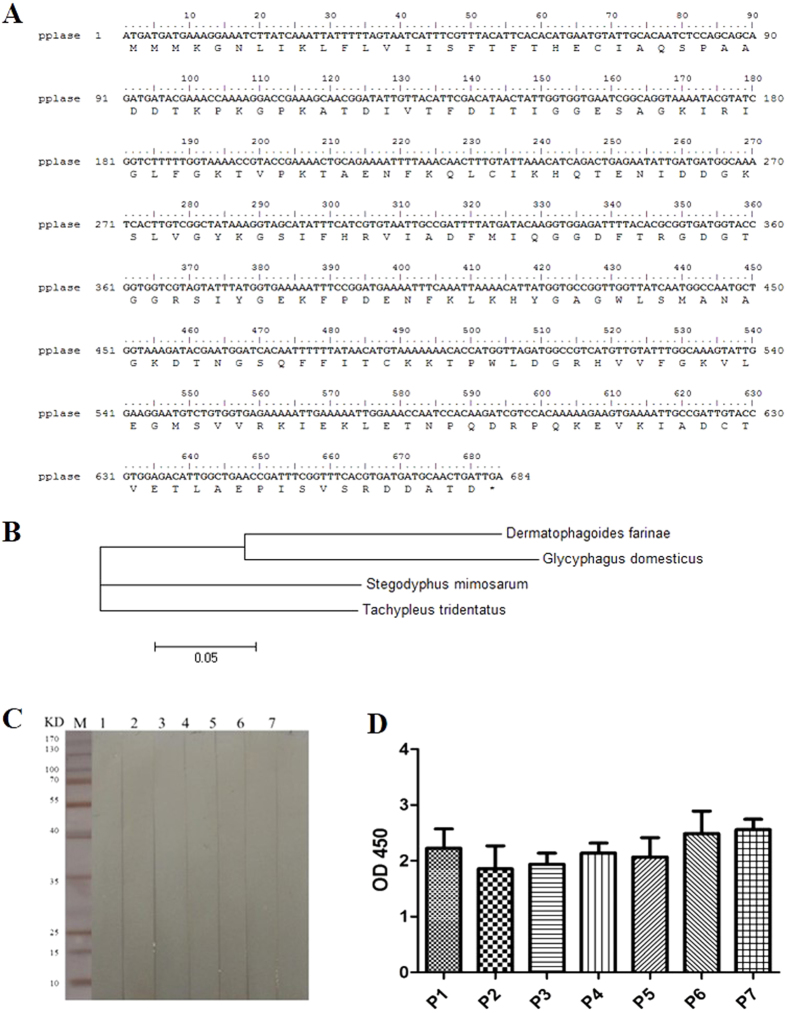
Immunological characterization of *D. farinae* Pplase. (**A**) *Pplase* sequence. (**B**) Evolutionary tree of *Der. farinae Pplase.* (**C**) Immunoblotting analysis of specific IgE reactivity to Pplase: 1–2, serum from healthy subjects; 3–7, serum from DME-positive patients. (**D**) Specific IgE reactivity to Pplase determined via ELISA: P1-P2, serum from healthy subjects; P3-P7, serum from DME-positive patients.

Pplase was cloned, expressed and purified (Fig. 1A,B). The allergenicity of Pplase showed no significant difference between asthma patients and healthy controls (Fig. 1C,D). The SPT results showed no positive response to Pplase protein. These findings indicated that Pplase is not an one of the allergens of *D. farinae* (Table 1).

### Pplase enhances allergic airway responses

**Figure 2:**
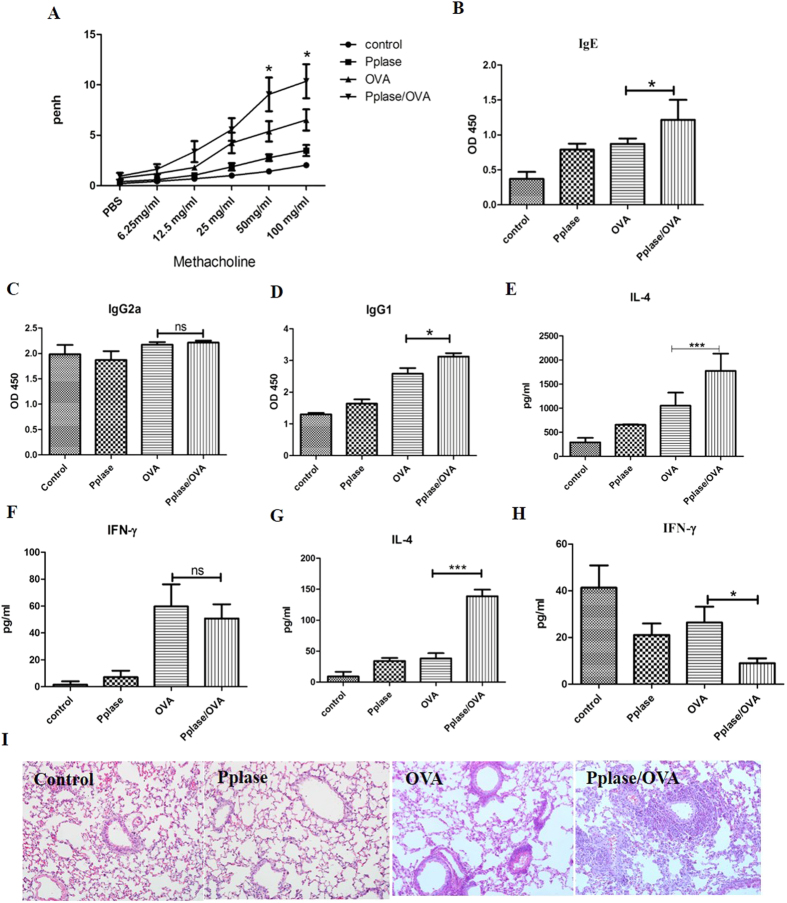
Pplase enhanced the development of airway pathology induced by OVA. Female BALB/C mice (6–8 weeks) were immunized by PBS, Pplase, OVA, or Pplase/OVA and then challenged with OVA or Pplase for one week. (**A**) Noninvasive measurement of AHR. Serum levels of allergen-specific (**B**) IgE, (**C**) IgG2a and (**D**) IgG1 were detected via ELISA as the optical density (OD). (**E**) and (**F**) were from the BALF; (**G**) and (**H**) were from the spleen. Single-cell suspensions from spleens were cultured for 72 hours in the presence of OVA or Pplase. (**I**) Histological sections of lung tissue from animals challenged with PBS, Pplase, OVA and Pplase/OVA. The data are shown as the mean ± SEM (n = 6) from one of 3 experiments. Significant differences between two groups were tested with an unpaired two-tailed t-test. *P < 0.05. ***P < 0.001. ns, no significant difference.

To further characterize Pplase, mice were immunized with PBS, Pplase, OVA, or Pplase/OVA (Fig. S1A). In the AHR experiment, we found that the Pplase groups showed no significant difference compared with the control groups, while a significant difference was observed between the OVA group and the control group. The Pplase/OVA group showed a stronger AHR than the OVA group, suggesting that Pplase promotes allergic responses to OVA (Fig. 2A). As shown in Fig. 2B–D, serum OVA-specific IgE and IgG1 levels were significantly higher in the Pplase/OVA group than the OVA group, but IgG2a antibody levels did not differ between the groups. As shown in Fig. 2E,F, the Pplase/OVA group presented higher levels of IL-4 than the OVA group. There was no difference in the expression of IFN-γ between the Pplase/OVA and OVA groups. The level of IL-4 in the supernatant of the spleen cell culture was consistent with that observed in BALF (Fig. 2G), but the levels of IFN-γ in the Pplase/OVA groups were lower than in the OVA groups. Lung tissue pathology was assessed, and we observed that more severe pathological changes were induced in the Pplase/OVA group than in the OVA group (Fig. 2I). Taken together, these data suggested that Pplase promotes the OVA-induced airway allergy and Th2 response.

### Pplase regulates DC activity via TLR4

**Figure 3:**
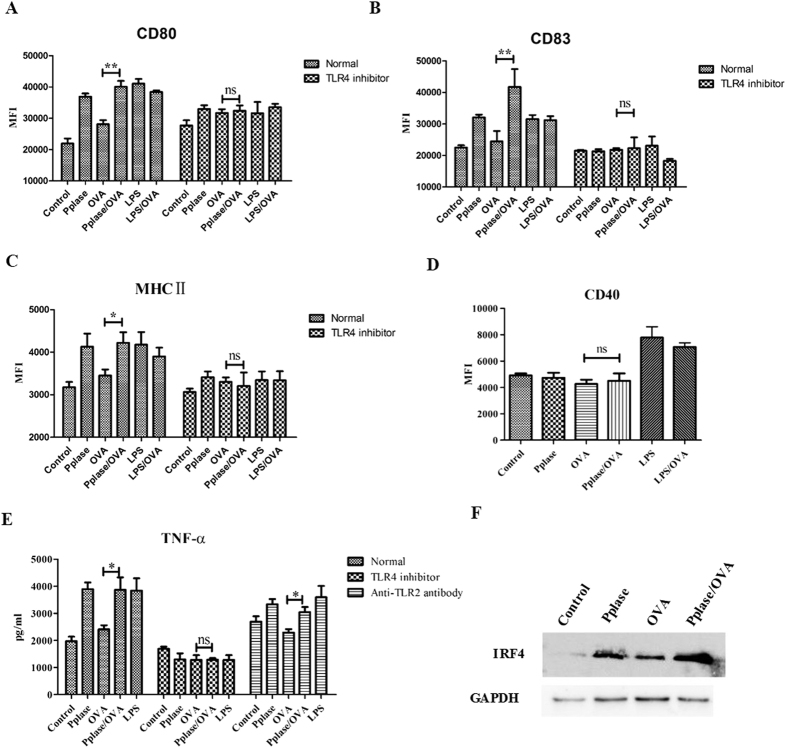
DCs were activated by Pplase via TLR4. DC2.4 cells were treated with Pplase, OVA, Pplase/OVA, LPS and LPS/OVA for 24 hours, and CD80, CD40, CD83 and MHC II levels were then detected via flow cytometry. (**A**) CD80 levels. (**B**) CD83 levels. (**C**) MHC II levels. (**D**) CD40 levels. DC2.4 cells were treated with or without CLI-095 for 6 h or an anti-TLR2 antibody for 2 h, and the secretion of TNF-α was detected via ELISA after 24 h. (**E**) Cytokine levels in the culture supernatants. The levels of IRF4 protein were detected using western blotting analyses. (**F**) Western blotting results. These results are expressed as the mean ± SEM from three different experiments. Significant differences between two groups were tested using a two-tailed t-test. *P < 0.05. **P < 0.01. ns, no significant difference.

To elucidate how Pplase enhances the Th2 immune response induced by OVA, the levels of IRF4, CD80, CD83, MHCII and CD40 in DCs were assessed. Using flow cytometry, we found that Pplase elevated the cell-surface expression of CD80, CD83 and MHCII but not CD40 (Fig. 3A–D). The results also showed that Pplase/OVA upregulated the gene expression of IRF4 in DCs compared with the OVA group (Fig. S2B). As shown in Fig. 3F, the IRF4 protein was upregulated by Pplase. The TNF-α level in DCs in the Pplase/OVA group was also significantly higher than in the OVA group (Fig. 3E). The mRNA level of TNF-α was significantly upregulated in the Pplase/OVA group (Fig. S2A), suggesting that Pplase could affect downstream signal transduction via cell-surface receptors and subsequently affect the transcription of cytokines. TLRs play an important role in allergic diseases, and many allergens affect the function of DCs by activating TLRs^10^. To further understand the potential pathways associated with Pplase, we detected molecules related to the TLR pathway using RT-PCR and found that the mRNA expression of TLR and NFκB was upregulated in DCs (Fig. S2C), suggesting that Pplase can bind to TLR to trigger downstream signaling pathways. To identify the Pplase receptor(s), inhibitors of TLR4 and TLR2 were used in the experiments. The results showed that the expression of TNF-α was suppressed in the presence of TLR4 signaling inhibitors, while there was no effect in the presence of an anti-TLR2 antibody compared with the control groups (Fig. 3E). Furthermore, the addition of TLR4 signaling inhibitors abolished the effect of Pplase on DCs (Fig. 3A–C).

### Pplase enforces Th2 polarization induced by OVA

**Figure 4:**
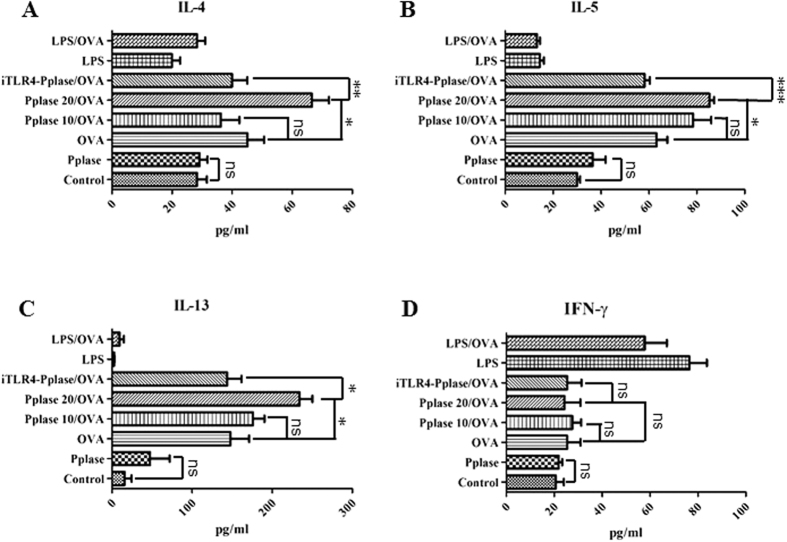
Pplase enhanced the Th2 polarization induced by OVA. BMDCs were co-cultured with spleen cells in the presence of PBS, Pplase (20 μg/ml), OVA (20 μg/ml), LPS (10 μg/ml), LPS (10 μg/ml)/OVA, Pplase10 (10 μg/ml)/OVA or Pplase 20 (20 μg/ml)/OVA, and after 5 days, cytokine levels were detected through ELISA. (**A**) IL-4, (**B**) IL-5, (**C**) IL-13 and (**D**) IFN-γ levels in the culture supernatants. The data were generated from three independent experiments. The significant differences between the two groups were tested using a two-tailed t-test. *P < 0.05. **P < 0.01. ***P < 0.001. ns, no significant difference.

On the basis of the above results, we proposed that Pplase directly activated DCs to enforce the Th2 polarization induced by OVA. To test this hypothesis, BMDCs from naïve BALB/C mice were co-cultured with spleen cells in the presence of OVA or Pplase. After 5 days, the supernatants were collected to measure cytokine levels via ELISA. As shown in Fig. 4A–D, only Pplase-treated BMDCs (Pplase) could not induce Th2 polarization, and OVA-plus, 10 μg/ml Pplase-treated BMDCs (Pplase 10/OVA) showed no significant differences compared with the OVA-only groups (OVA). However, OVA-plus, 20 μg/ml Pplase-treated BMDCs (Pplase 20/OVA) enhanced IL-5, IL-4 and IL-13 expression levels compared with the OVA groups. Moreover, LPS-treated BMDCs (LPS) and OVA-plus, LPS-treated BMDCs (LPS/OVA) exhibited dramatically elevated IFN-γ levels, while the Th2 cytokine level did not differ from the control groups. In addition, in the presence of TLR4 signaling inhibitors (iTLR4-Pplase/OVA), Pplase-treated BMDCs did not enhance Th2-cytokine levels.

### Allergic airway diseases induced by OVA are enhanced by adoptive transfer with Pplase-primed BMDCs

**Figure 5:**
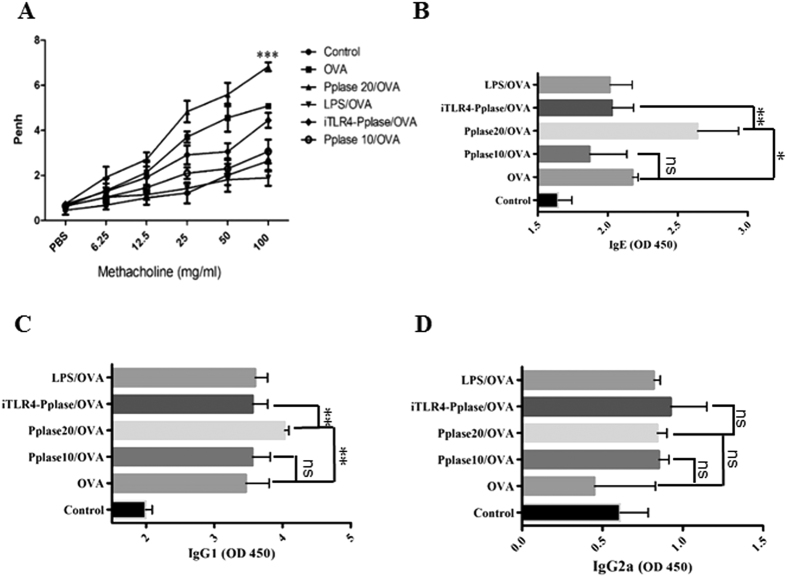
Pplase-treated BMDCs enhanced airway allergy following transfer into previously sensitized and challenged recipients. BMDCs were pre-cultured in the presence of PBS, Pplase (20 μg/ml), LPS (10 μg/ml) or Pplase (10 μg/ml). After 24 hours, the cells were collected for transfer into female BALB/C mice (6–8 weeks) via intravenous tail injection prior to OVA priming, and an asthma mouse model was generated. (**A**) Noninvasive measurement of AHR. Serum allergen-specific (**B**) IgE, (**C**) IgG1 and (**D**) IgG2a levels were detected via ELISA as the optical density (OD). The data are shown as the mean ± SEM (n = 6). The significant differences between two groups were tested with a two-tailed t-test. *P < 0.05. **P < 0.01. ***P < 0.001. ns, no significant difference.

**Figure 6:**
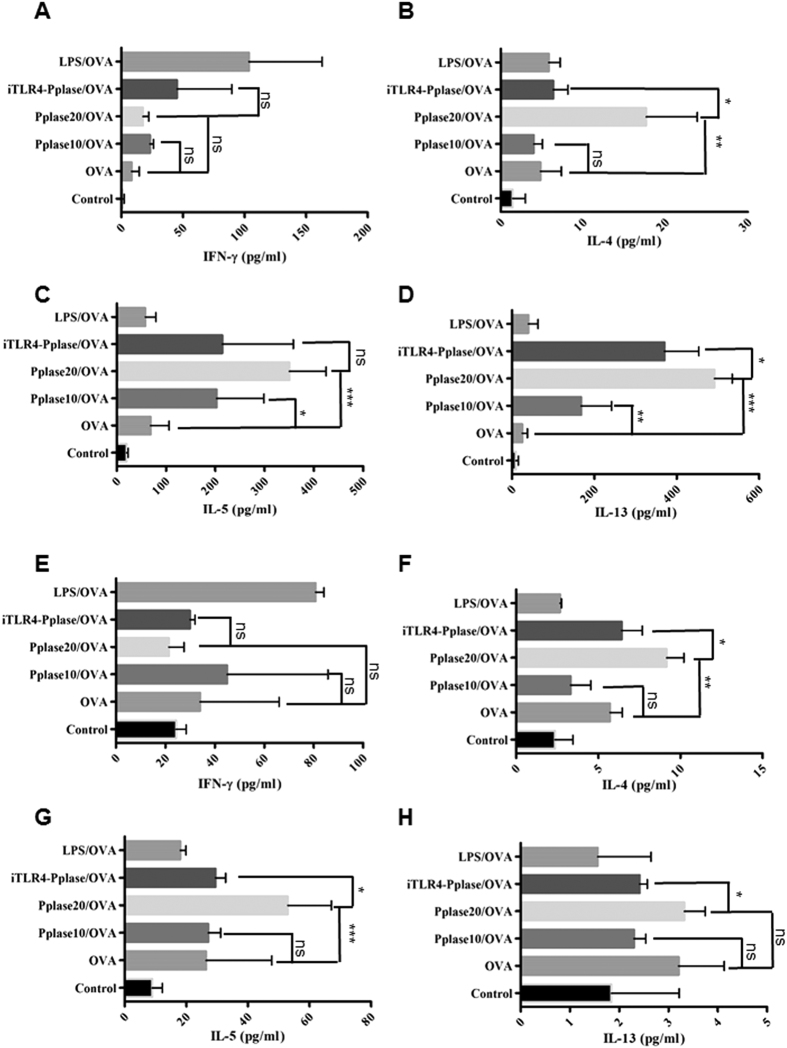
Transfer of Pplase-treated BMDCs enhanced Th2 immune responses induced by OVA. BMDCs were pre-cultured in the presence of PBS, Pplase (20 μg/ml), LPS (10 μg/ml) or Pplase (10 μg/ml). After 24 hours, the cells were collected for transfer into female BALB/C mice (6–8 weeks) via intravenous tail injection prior to OVA priming, and an asthma mouse model was generated. Cytokines from the supernatants of the spleen cell cultures and BALF were tested using ELISA. (**A–D**) show results for the supernatants of spleen cell cultures. (**E–H**) show results for BALF. The data are shown as the mean ± SEM (n = 6). Significant differences between two groups were tested using a two-tailed t-test. *P < 0.05. **P < 0.01. ***P < 0.001. ns, no significant difference.

To further clarify the functions of Pplase, we transferred Pplase-primed BMDCs to naïve mice, and the mice were subsequently sensitized and challenged with OVA (Fig. S1B). As shown in Fig. 5A–D, recipients of 20 μg/ml Pplase-primed BMDCs (Pplase 20/OVA) showed higher AHR, serum IgE and IgG1 than the recipients of vehicle-primed BMDCs. The levels of IL-4, IL-5 and IL-13 in the supernatant of spleen cell cultures and BALF were also upregulated in the Pplase 20/OVA groups (Fig. 6A–H). However, transfer of a lower concentration of Pplase-primed BMDCs (Pplase 10/OVA) resulted in the same response observed in the OVA group (Fig. 6A–H). In addition, recipients of Pplase-primed BMDCs treated with TLR4 signaling inhibitors (iTLR4-Pplase/OVA) showed a significantly lower AHR and Th2 immune responses than the Pplase 20/OVA groups (Fig. 6A–H). Moreover, there was no difference in the expression of IFN-γ and IgG2a between these groups (Figs 5D and 6A,E). Recipients of LPS-treated BMDCs (LPS/OVA) exhibited elevated IFN-γ levels and reduced AHR compared with the OVA groups (Figs 5A and 6A,E).

## Discussion

Allergens from dust mites play an important role in the pathogenesis of asthma. The rate of positivity for HDMs is as high as 70–80% in asthma patients^24^, while published data have indicated that the rates of positivity for allergens from species other than dust mites are generally lower than 40% in asthma patients^24^. The therapeutic effect of a dust mite crude extract on asthma is greater than that of recombinant mite allergens observed under specific immunotherapy^25^, suggesting that the effect of dust mites in the pathogenesis of asthma may be different from that of other allergens. In previous studies, we identified *Pplase* by sequencing the dust mite genome^15^. In the present study, we assessed the allergenicity of Pplase. The results of this work showed that the dust mite Pplase protein is not an allergen. However, the data revealed that the Pplase protein could enhance allergic responses induced by OVA. We propose that there may be many other non-allergen proteins present in *D. farinae*, which could facilitate the development of polarizing Th2 immune responses, thus potentially accounting for the high positive rates for dust mites in asthma patients.

CD4+ T helper cells play an important role in the pathogenesis of asthma by secreting IL-4, IL-5 and IL-13^26^. DCs, which are the most important type of APC, interact with T cells to elicit T cell activation through multiple cell-surface interactions^27^. Activation of CD4+ T lymphocytes requires not only antigen signals but also costimulatory molecular stimuli, and the latter may be a major determinant of eliciting an immune response or immune tolerance^28^,^29^. CD80/CD86 on the surface of APCs have been intensively studied; these proteins interact with CD28 and cytotoxic T lymphocyte–associated antigen 4 (CTLA) and are regarded as the predominant factor in the pathogenesis of allergic asthma^30^. In this study, the expression of CD80, CD83 and MHCII on the surface of DCs was found to be significantly upregulated by Pplase.

IRF4 plays an important role in the differentiation and function of T cells and B cells^31^,^32^. In a *Leishmania* infection model, knockout of the IRF4 gene in DCs can significantly reduce parasitic infection and upregulate IFN-γ and IL-12, suggesting that the IRF4 of DCs may inhibit the Th1 responses induced by *Leishmania*^33^. In a *Nippostrongylus brasiliensis* infection model, the CD11c+ cells of IRF4 knockout mice show a significantly lower number of IL-4-, IL-5-, and IL-13-producing CD4+ T cells, suggesting that IRF4 expression by DCs is important for inducing Th2 responses^34^. The evidence suggests that IRF4 from DCs can drive Th0 cells toward differentiation into Th2 cells. IRF4 was shown to be upregulated by Pplase in DC2.4 cells, indicating that the Pplase protein of dust mites can enhance IRF4 expression.

TLRs distributed on the surface of DCs can identify highly conserved pathogen motifs to regulate the function of DCs^11^,^35^. Epidemiological surveys have shown that TLR2 and TLR4 gene polymorphisms are positively correlated with human allergic asthma^36^,^37^,^38^. Previous studies have shown that *D. pteronyssinus* crude extract and Der P2 induce allergic asthma via the TLR2 signaling pathway, and TLR4 also plays an important role in allergic disease induced by HDMs^39^,^40^. In the present study, TLR4 was suggested to act as a receptor of Pplase, and the CD80, CD83, MHCII and TNF-α phenotypes were reduced in DCs after blockade of TLR4.

On the basis of our results, we hypothesized that Pplase enhances allergic diseases mainly by regulating the functions of DCs. Our data showed that Th2 cell differentiation is not induced by Pplase alone but indicated that Pplase does facilitate Th2 polarization induced by OVA. This hypothesis was corroborated by *in vivo* experiments, and adoptive transfer with Pplase-primed DCs facilitated the development of airway allergy induced by OVA, which was abolished by the blockade of TLR4 signals. These results suggested that Pplase mainly ligated TLR4 to activate DCs, thereby promoting the development of airway allergy induced by allergens.

In summary, although the Pplase protein is not an allergen of *D. farinae*, it can enhance allergic disease induced by other allergens.

## Additional Information

**How to cite this article:** Wang, H. *et al*. Pplase of *Dermatophagoides farinae* promotes ovalbumin-induced airway allergy by modulating the functions of dendritic cells in a mouse model. *Sci. Rep.* **7**, 43322; doi: 10.1038/srep43322 (2017).

**Publisher's note:** Springer Nature remains neutral with regard to jurisdictional claims in published maps and institutional affiliations.

## Supplementary Material

Supplementary Figure S1 and S2

## Figures and Tables

**Table 1:** Results of the skin prick tests (SPT).

No	Gender/Age	Diagnosis	Net wheal size (mm), Level			
			DME	Histamine	NS	Pplase
1	Male/47	BA, AR	3.5, +++	5.5	0	0
2	Male/45	AR	2, +++	7.5	0	0
3	Female/52	AR, FA	1.5, +	5.5	0	0
4	Male/20	BA, FA	1.5, +	5	0	0
5	Female/20	BA	2, ++	4	0	0
6	Female/13	AR	1.25, ++++	6	0	0
7	Female/66	BA, AR, DA	9, +++	5.5	0	0
8	Female/64	BA	2.25, +	6	0	0
9	Female/41	BA, AR	3.5, ++	5.5	0	0
10	Female/44	BA, AR	4.5, +++	4.5	0	0

AR (Allergic rhinitis); BA (Bronchial asthma); FA (Food allergy); DA (Drug allergy); DME (Dust mite extract); NS (Normal saline). Positive: ≧ 1; Negative: 0.
