# Supplementary material for: Pplase of Dermatophagoides farinae promotes ovalbumin-induced airway allergy by modulating the functions of dendritic cells in a mouse model
Source: Sci Rep. 2017 Feb 27;7:43322. doi: 10.1038/srep43322 (PMC5327411; doi:10.1038/srep43322)
Supplement: Supplementary Figure S1 and S2 [file srep43322-s1.doc]

**Supplementary materials**

**Pplase of *Dermatophagoides farinae* promotes** **ovalbumin-induced airway allergy by modulating the functions of dendritic cells in a mouse model**

**Running title**: Pplase facilitates allergic diseases

Hui Wang^1#^, Lihua Mo^1, 2#^, Xiaojun Xiao^1#^, Shu An^3^, Xiaoyu Liu^1^, Jinge Ba^1^, Weifang Wu^1^, Pixin Ran^4^, Pingchang Yang^1, 2^ and Zhigang Liu^1, 2, 3*^

^1^ State Key Laboratory of Respiratory Disease for Allergy at Shenzhen University, Shenzhen Key Laboratory of Allergy & Immunology, Shenzhen University School of

Medicine, Shenzhen, China.

^2^Shenzhen ENT Institute, Longgang ENT Hospital, Shenzhen, China.

^3^Luohu district people's hospital, Shenzhen, China.

^4^State Key Laboratory of Respiratory Disease, Guangzhou Medical University, Guangzhou 510006, China.

^#^These authors equally contributed to this work.

**Corresponding authors**:

Dr. Zhigang Liu and Dr. Pingchang Yang. Shenzhen University. Room 722 of Medical School Bldg., 3688 Nanhai Blvd, Shenzhen, 518060 China. Tel: 8675526681907. Fax: 8675526681906. E-mail: lzg@szu.edu.cn and [pcy2356@szu.edu.cn](mailto:pcy2356@szu.edu.cn)

Dr. Pixin Ran, State Key Laboratory of Respiratory Disease, Guangzhou Medical College, Guangzhou 510006, China. E-mail: [pxran@vip.163.com](mailto:pxran@vip.163.com)

**Supplemental figures**


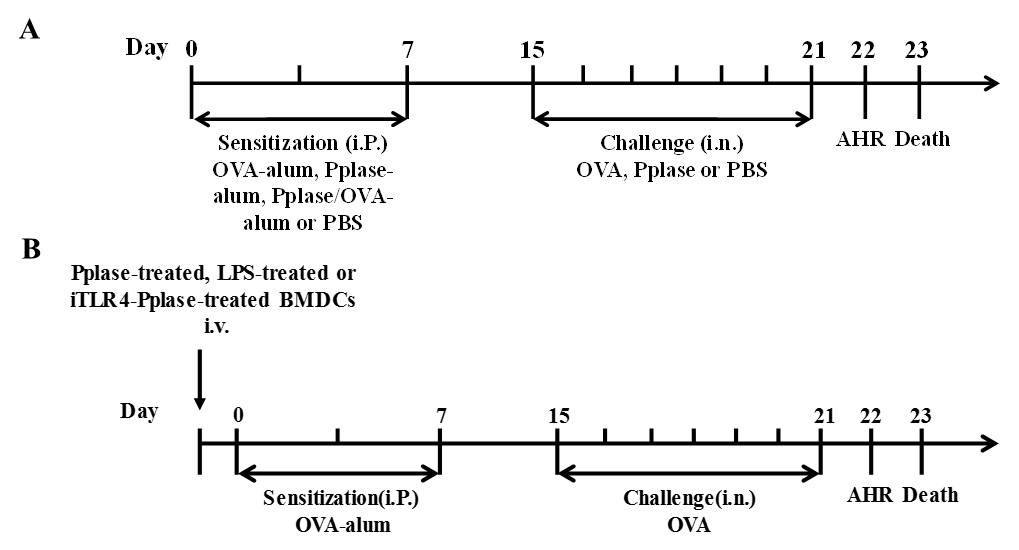


**Figure S1. The scheme of establishment of mouse model.**

**Figure S2.** **The DCs were activated by Pplase via TLR4.**

DC2.4 cells were stimulated with PBS, Pplase, OVA and Pplase/OVA at 20μg/ml, following a 24-hour stimulating, the gene expression were detected by RT-PCR. (A)TNF-α levels; (B) IRF4 expression levels; (C) TLR-associated genes levels.
